# Supplementary material for: Genetic and Real-World Clinical Data, Combined with Empirical Validation, Nominate Jak-Stat Signaling as a Target for Alzheimer’s Disease Therapeutic Development
Source: Cells. 2019 May 8;8(5):425. doi: 10.3390/cells8050425 (PMC6562942; doi:10.3390/cells8050425)
Supplement: Supplementary file 1 [file cells-08-00425-s001.zip › Fig s1.docx]

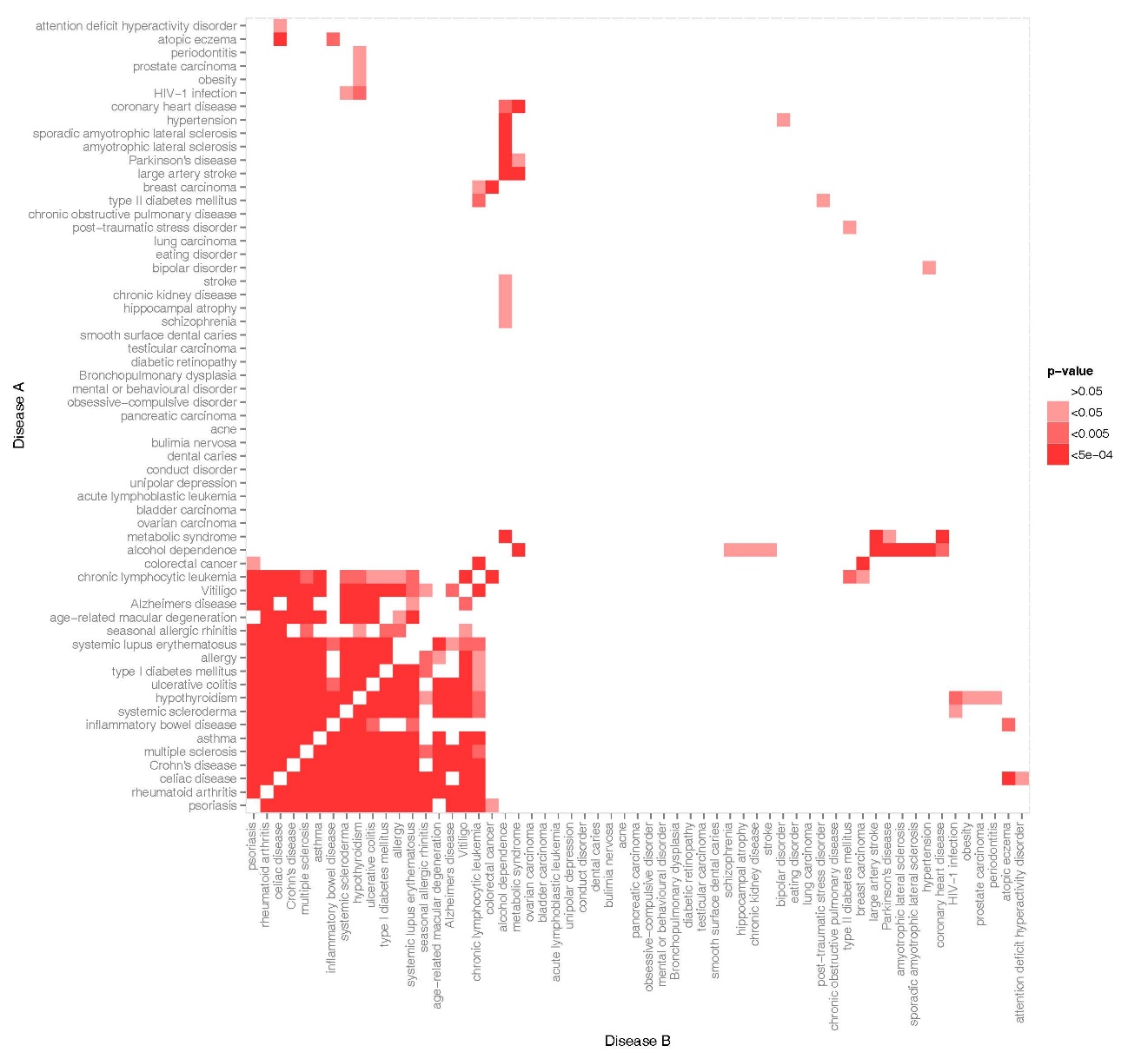


Fig. S1. Overlap of GWAS genes, full version.

This graph represents the full version of Fig. 2, now showing all 59 analyzed diseases. As in Fig. 2, the colour of each square in the table represents the p-value obtained when calculating whether the GWAS-genes of diseases A and B overlap in the pathway space more than would be expected from chance.
